# *Cetobacterium* alleviates the lipid accumulation in Nile tilapia (*Oreochromis niloticus*) induced by leucine addition

**DOI:** 10.3389/fmicb.2025.1708010

**Published:** 2026-01-16

**Authors:** Miao Wang, Chenglin Tang, Zhigang Liu, Jianmeng Cao, Zhang Wang, Maixin Lu, Mengmeng Yi, Xiaoli Ke

**Affiliations:** ^1^Pearl River Fisheries Research Institute, Chinese Academy of Fishery Science, Guangzhou, China; ^2^Key Laboratory of Tropical and Subtropical Fishery Resource Application and Cultivation, Ministry of Agriculture and Rural Affairs, Guangzhou, China; ^3^Guangdong Provincial Key Laboratory of Aquatic Animal Immunology and Sustainable Aquaculture, Guangzhou, China

**Keywords:** *Cetobacterium*, insulin, leucine, lipid, tilapia

## Abstract

Deviation from the optimal dietary leucine range has been shown to have negative effects on fish growth and metabolism. This study investigated the dose-dependent effects of dietary leucine on lipid metabolism in Nile tilapia (*Oreochromis niloticus*). Four diets were formulated: a control diet containing the basal leucine level (2.3% of diet) and three test diets supplemented with 0.8, 1.6%, or 2.4% leucine, respectively. After 12 weeks of feeding, fish receiving ≥ 0.8% supplemental leucine showed significant growth inhibition (lower body weight and total length) and metabolic disorders, including hyperinsulinaemia and elevated serum total cholesterol (TC) and triglycerides (TG) levels, together with increased hepatic lipid-droplet area. Tilapia in the 2.4% leucine-addition group exhibited a markedly higher hepatosomatic index (HSI), whereas blood glucose was significantly reduced in tilapia of the 1.6 and 2.4% leucine-addition groups. Liver transcriptome comparison (2.4% leucine-addition vs. control) revealed activation of amino acid synthesis and the mTOR signaling pathway. qRT-PCR confirmed that excessive leucine up-regulated the expression of key lipogenic genes, such as *IRS1* and *ACC*. 16S rRNA sequencing further revealed a 2.3-fold increase in the relative abundance of *Cetobacterium* in the intestine of the 2.4% leucine-addition group. Subsequent intervention with *Cetobacterium somerae* NK01 significantly reversed leucine-induced metabolic disorders, with a decrease in serum TC and TG levels by 7.8 and 10%, respectively, and a 58.3% reduction in hepatic lipid-droplet area. These beneficial effects were associated with the modulation of lipid-related genes, including *IRS1*, *PI3K*, *SREBP1c*, *ACC*, and *FAS*. Collectively, the data demonstrated a dual role of leucine in tilapia lipid metabolism: it promoted lipid lipogenesis by activating the mTOR-SREBP1c signaling axis while simultaneously enriching the intestinal symbiont *Cetobacterium* to alleviate metabolic stress.

## 1 Introduction

After a glucose load, fish recover slowly to basal glycaemia and thus exhibit prolonged hyperglycemia (Polakof et al., 2012). Although circulating insulin concentrations in teleost are generally higher than mammals, insulin sensitivity is low and glucose tolerance is considered poor (Moon, 2001). High blood sugar can lead to lipid deposition and oxidative stress in fish (Li X. et al., 2021).

Nile tilapia (*Oreochromis niloticus*) has become a globally farmed species owing to its rapid growth, hypoxia tolerance, and wide environmental adaptability (El-Sayed and Fitzsimmons, 2023). In commercial culture, however, the fish frequently develop metabolic disorders, most notably excessive hepatic and visceral fat deposition. This problem stems from its limited capacity to digest and utilize dietary carbohydrate. When glucose supply excesses demand, the surplus is channeled into de-novo lipogenesis in the liver, resulting in pathological fat accumulation in liver and other organs (Wilson, 1994). Fatty liver syndrome jeopardizes production by depressing growth, lowering feed conversion, increasing cost, impairing immunity and stress tolerance, and elevating mortality (Du, 2014).

Studies in murine models have revealed that leucine exerts complex, nutrition-dependent effects on lipid metabolism. Under a high-fat dietary background, leucine supplementation shows multiple metabolic benefits. Freudenberg et al. (2013) found that L-leucine supplementation markedly lowered hepatic triglyceride (TG) level in high-fat diet-fed mice and down-regulated fatty acid synthase gene expression. Zhang et al. (2007) further showed that leucine raised energy expenditure by up-regulating uncoupling protein 3 (UCP3), improved insulin sensitivity, and alleviated high-fat diet-induced metabolic disorders. These benefits were largely absent under a low-fat diet background (Noatsch et al., 2011), indicating that leucine’s metabolic actions were tightly linked to whole-body energy status.

In fish, requirement studies based on weight gain and feed efficiency placed the optimal dietary leucine level at 0.8–3.29% of dry diet (Gan et al., 2016; Ren et al., 2015; Tan et al., 2016; Yang et al., 2022). Deviation from the optimal range will adversely affect the fish growth and metabolism (Gan et al., 2016; Liang et al., 2019). Previous studies indicated that the level of dietary leucine was closely related to fish lipid metabolism. Increasing leucine content can up-regulate the expression of lipid synthesis genes, increase serum TG concentration and raise carcass fat content. For example, in studies on fingerling *Catla catla* (Hamilton) (Zehra and Khan, 2015) and fingerling *Cirrhinus mrigala* (Hamilton) (Ahmed and Khan, 2006), carcass fat demonstrated a positive association with increasing leucine concentration in the diet. Likewise, blunt-snout bream (*Megalobrama amblycephala*) fed 1.33% leucine displayed higher serum TG and hepatic *FAS* (fatty acid synthase) expression than fish fed 0.90% leucine (Liang et al., 2019). However, the mechanisms underlying these effects remained unclear and warranted further investigation.

In recent years, growing attention was being paid to the interaction between gut microbiota and host metabolism. The gut microbiota substantially influenced the host’s amino-acid metabolism, offering a new lens through which to examine nutrient-microbe-host interactions. In finishing pigs, leucine supplementation decreased body-fat mass and altered gut microbial composition (Hu et al., 2019). Based on the above research background, the present study aimed to explore the role of leucine in regulating tilapia lipid metabolism from the perspective of gut microbiota.

## 2 Materials and methods

### 2.1 Experiment design

#### 2.1.1 Dietary leucine supplementation trial

This study was approved by the Laboratory Animal Ethics Committee of Pearl River Fisheries Research Institute, Chinese Academy of Fishery Sciences, and followed the Animal Welfare Guidelines of China. According to Xu et al. (2022), dietary supplementation with 2.3% leucine resulted in the best growth performance and hepatic health in Nile tilapia. To investigate how leucine modulates lipid metabolism, we added four incremental levels of leucine (0, 0.8, 1.6, 2.4%) on top of the basal 2.3% leucine diet (Supplementary Table S1). Four treatments (CK, LE1, LE2, LE3) were established, each with three replicate tanks (*n* = 3) of 30 juvenile tilapia (initial body weight: 29 ± 3.5 g). Fish were hand-fed twice daily at 4% of mean body weight. Water was continuously aerated without exchange under a 12 h L: 12 h D photoperiod. Water temperature was maintained at 27.8 ± 0.6 °C, pH at 7.30 ± 0.20, and dissolved oxygen (DO) at 5.80 ± 0.70 mg/L. Total nitrogen (TN), ammonia nitrogen (NH_4_-N), and nitrite nitrogen (NO_2_-N) concentrations were 0.24 ± 0.12, 0.15 ± 0.09 L, and 0.01 ± 0.007 mg/L, respectively.

After the 12-week trial, six fish per treatment (fasted 24 h) were randomly sampled and anaesthetized with MS-222 (100 mg/L). Total length, body length, body weight and liver weight were recorded; blood was withdrawn for serum collection; mid-gut contents were collected for 16S rRNA sequencing; liver was snap-frozen for transcriptome and qPCR. Serum glucose, TC, and TG were quantified using TC/TG/Glucose Assay Kit (Jiangsu Meimian). Insulin was measured with the Tilapia INS ELISA Kit (Mlbio, Shanghai). Growth indices including feed conversion ratio (FCR), hepato-somatic index (HSI), weight gain rate (WGR), specific growth rate (SGR), and condition factor (CF) were calculated as described by Du and Turchini (2021).

#### 2.1.2 Effects of *Cetobacterium somerae* NK01 on leucine levels and lipid metabolism in tilapia

A tilapia fatty-liver model was established using the dietary combination that produced the highest HSI. Three experimental groups were established: control group (CK: basal diet), fatty-liver model group (LE: basal diet + 2.4% leucine), and fatty-liver model + *C. somerae* NK01 group (LN: basal diet + 2.4% leucine + *Cetobacterium* NK01-supplement at a concentration of 1 × 10^7^ CFU/g diet). The bacterial dose was selected according to Qi et al. (2023) and Wang et al. (2025). Each group contained three biological replicates. Intestinal contents (mid-gut) were collected for short-chain fatty acids (SCFAs) analysis, and blood was withdrawn for serum amino-acid profiling and for analyzing lipid metabolism-related indices. Liver was snap-frozen for qPCR. Biochemical assays of serum were conducted, and growth parameters were determined followed Section 2.1.1.

### 2.2 Oil Red O staining

After air-drying frozen sections for 60 min, they were fixed in 10% neutral buffered formalin at 4 °C for 10 min. The fixative was discarded and the sections were rinsed twice with double-distilled water (ddH₂O), then air-dried at room temperature for 45 min. Sections were subsequently stained with pre-warmed Oil-Red-O working solution at 60–65 °C for 15 min and differentiated in 80% propylene glycol for 2–5 min. After three washes with ddH₂O, they were counterstained with hematoxylin for 40 s and rinsed in running water for 3 min. Finally, sections were examined under a light microscope. Lipid-droplet areas were quantified with Image-Pro Plus 6.0 (Media Cybernetics, Silver Spring, MD, United States); ten random fields per sample were measured to calculate the relative lipid areas.

### 2.3 Amino acid analysis of tilapia serum

Samples were extracted as described by Zhang et al. (2024). Standard solutions and extracts were analyzed using LC–MS. Target amino-acid concentration were calculated from the peak-area ratio of each analyte to its internal standard.

### 2.4 SCFAs analysis of tilapia gut content

Samples were prepared according to Fan et al. (2023). SCFA concentrations were determined by LC–MS using the peak-area ratio of each target analyte to the internal standard.

### 2.5 RNA isolation and Illumina sequencing

RNA extraction and Illumina sequencing were performed as described by Hou et al. (2023). Reads were mapped to the reference genome using HISAT2. Differential gene expression between groups was analyzed with DESeq2 algorithm. Kyoto Encyclopedia of Genes and Genomes (KEGG) pathway enrichment of differentially expressed genes was assessed with the KOBAS database (Mao et al., 2005) and clusterProfiler.

### 2.6 Quantitative real-time PCR

Quantitative real-time PCR (qRT-PCR) was performed following the protocol described by Hou et al. (2023). Primer sequences used in this study are listed in Supplementary Table S2.

### 2.7 Microbiota analysis of intestinal content

16S rRNA sequencing was conducted according to Wang et al. (2022). Raw sequences were processed utilizing the QIIME2 platform; ASVs were generated and taxonomically assigned. Alpha-diversity indices were calculated from the ASV table. Beta-diversity analysis (Bray–Curtis dissimilarity) was visualized by principal-coordinate analysis (PCoA). The taxonomic profiles were further depicted with MEGAN and GraPhlAn. Furthermore, random-forest classification was performed in QIIME2 to evaluate the potential of the microbiota to distinguish between groups.

### 2.8 Data analysis

The data were presented as the mean ± standard error. Statistical analysis was conducted using SPSS 24.0 (IBM, United States). The Shapiro–Wilk test was utilized to evaluate the normality of data distribution. Differences among groups were evaluated by One-Way ANOVA followed by Duncan’s test (equal variances) or Dunnett’s T3 test (unequal variances). Graphs were generated by SigmaPlot 12.0 (Systat Software, Germany).

## 3 Results

### 3.1 Dietary leucine modulates growth and serum biochemistry

**Figure 1:**
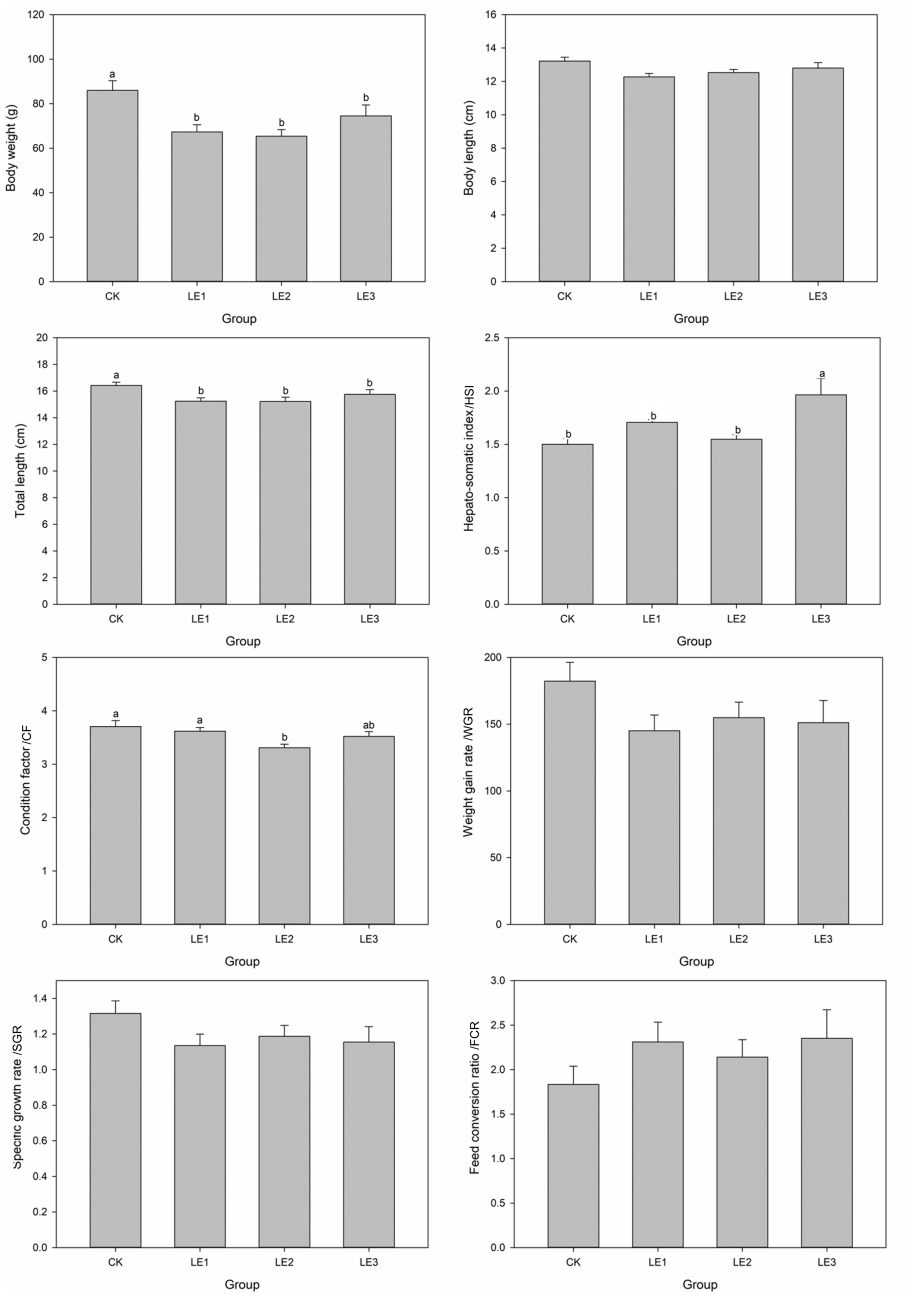
Growth performance parameters of tilapia fed diets containing different levels of leucine. CK: Control, LE1: 0.8% leucine addition, LE2: 1.6% leucine addition, and LE3: 2.4% leucine addition. Different lowercase letters indicate significant differences among groups for the same parameter (*p* < 0.05).

During the experiment, no mortality occurred in any of the groups. Analysis of body weight, total length, HSI, and CF revealed that dietary leucine induced physiological changes (Figure 1). Body weight and total length of tilapia in the CK group were significantly higher than those in the other three groups (*p* < 0.05). CF in the CK and LE1 groups was also significantly higher than that in LE2 group (*p* < 0.05). Body length, WGR, and SGR of tilapia in CK group were numerically greater than those in the other groups, but the differences were not statistically significant (*p* > 0.05). Moreover, HSI of tilapia in LE3 group was highest and differed significantly from the other three groups (*p* < 0.05).

**Figure 2:**
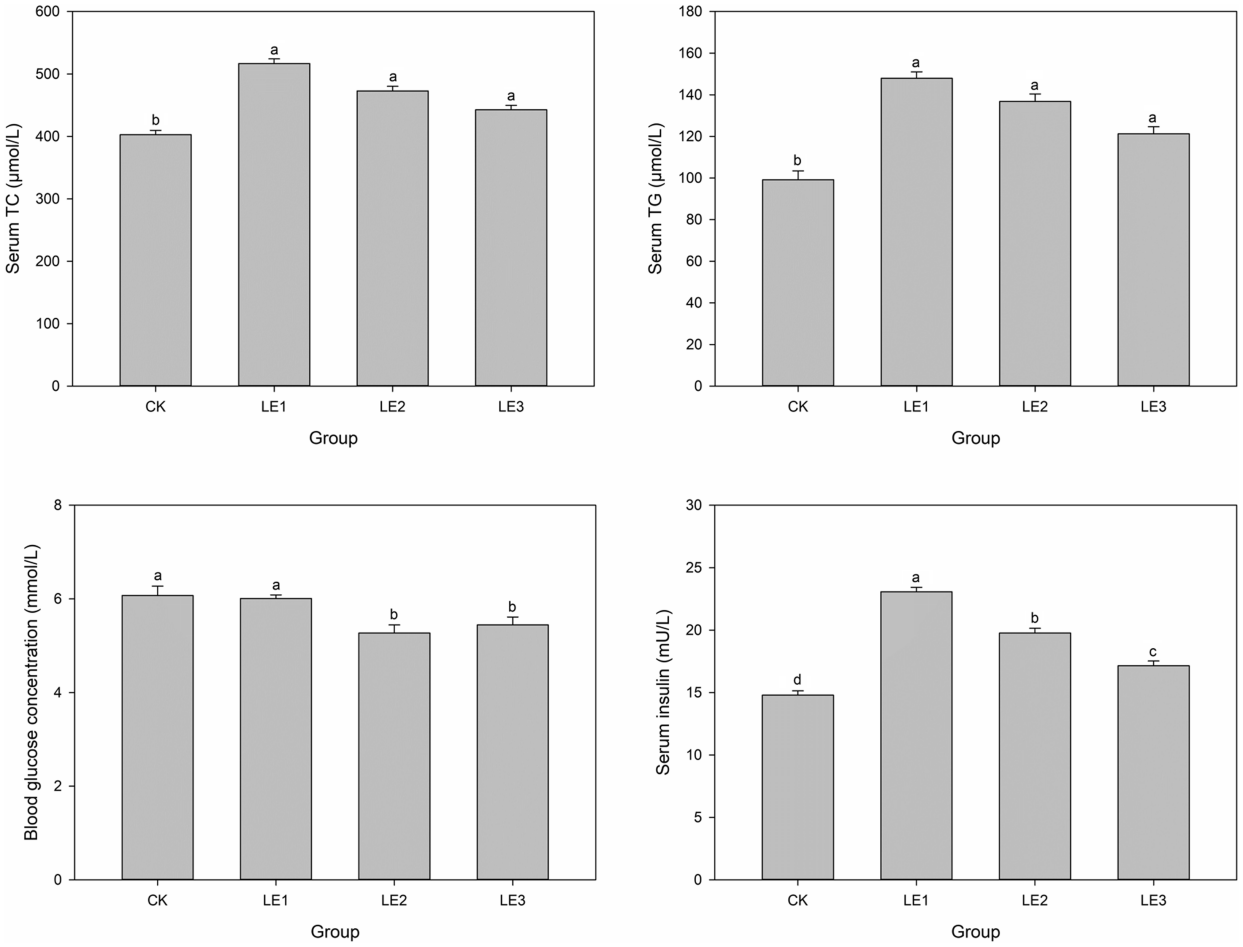
Levels of total cholesterol (TC), triglycerides (TG), glucose, and insulin in the serum of tilapia fed with diets containing different levels of leucine. CK: Control, LE1: 0.8% leucine addition, LE2: 1.6% leucine addition, and LE3: 2.4% leucine addition. Different lowercase letters indicate significant differences among groups for the same parameter (*p* < 0.05).

Serum TC, TG and insulin levels in CK were significantly lower those than in the other three groups (*p* < 0.05) (Figure 2). Blood glucose concentration of tilapia in LE2 and LE3 was significantly lower than that in CK and LE1 groups (*p* < 0.05).

### 3.2 Leucine induces hepatic steatosis

**Figure 3:**
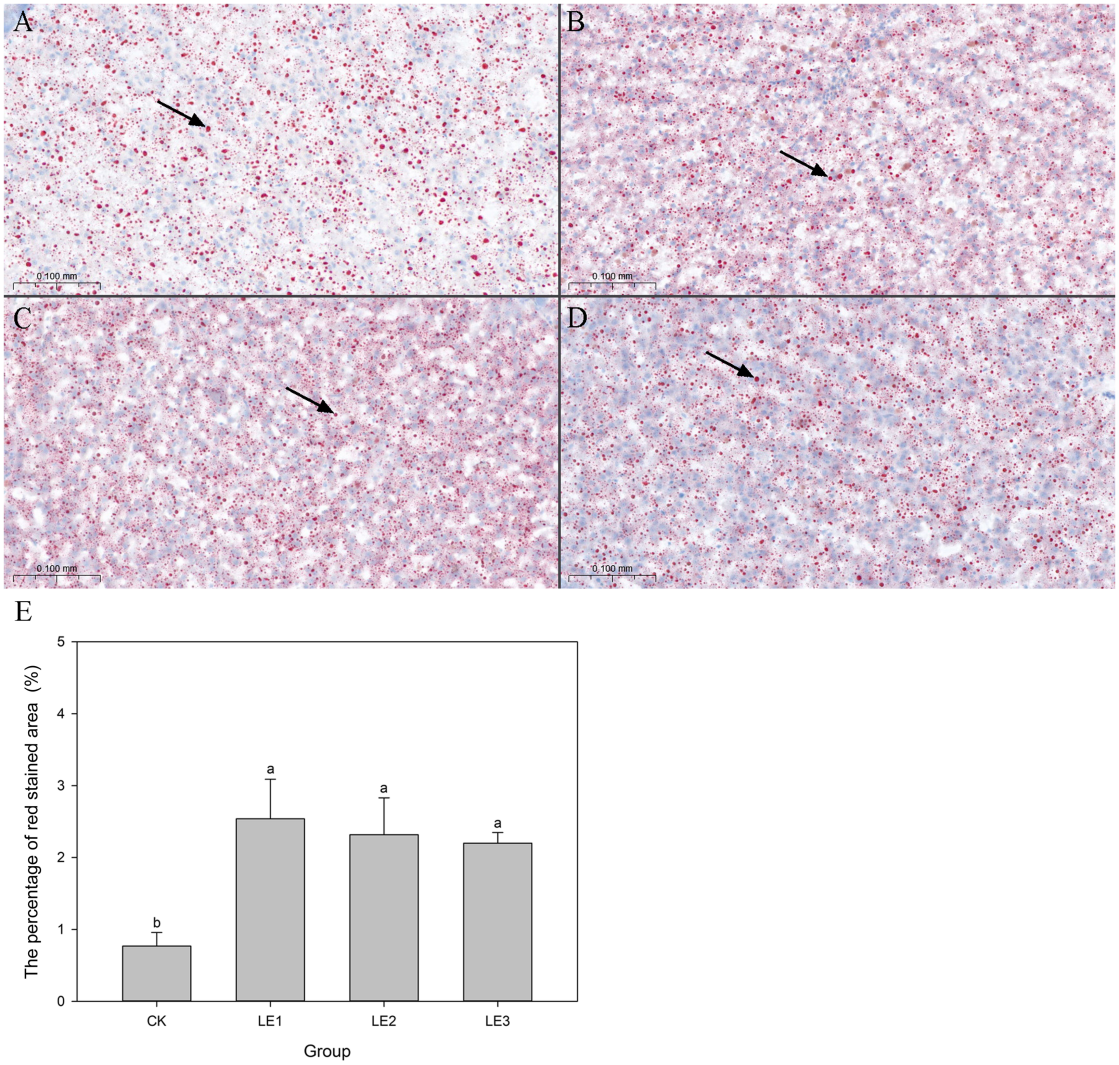
Oil-Red O staining of hepatic lipid droplets in tilapia fed diets containing different levels of leucine. **(A)** Oil-Red O staining of hepatic lipid droplets in the CK group. **(B)** Oil-Red O staining of hepatic lipid droplets in the LE1 group. **(C)** Oil-Red O staining of hepatic lipid droplets in the LE2 group. **(D)** Oil-Red O staining of hepatic lipid droplets in the LE3 group. **(E)** The percentage of red stained area quantified utilizing ImageJ Pro Plus 6 software. Original magnification: 40×; scale bars: 0.05 mm. Lipid droplets are displayed in red; nuclei in blue. CK: Control, LE1: 0.8% leucine addition, LE2: 1.6% leucine addition, and LE3: 2.4% leucine addition.

Oil-Red-O staining confirmed lipid accumulation in livers of all groups (Figures 3A–D). Moreover, the relative lipid-droplet area of liver in LE1, LE2, and LE3 groups was significantly higher than that in CK group (*p* < 0.05) (Figure 3E).

### 3.3 Transcriptional regulation of hepatic metabolism

**Figure 4:**
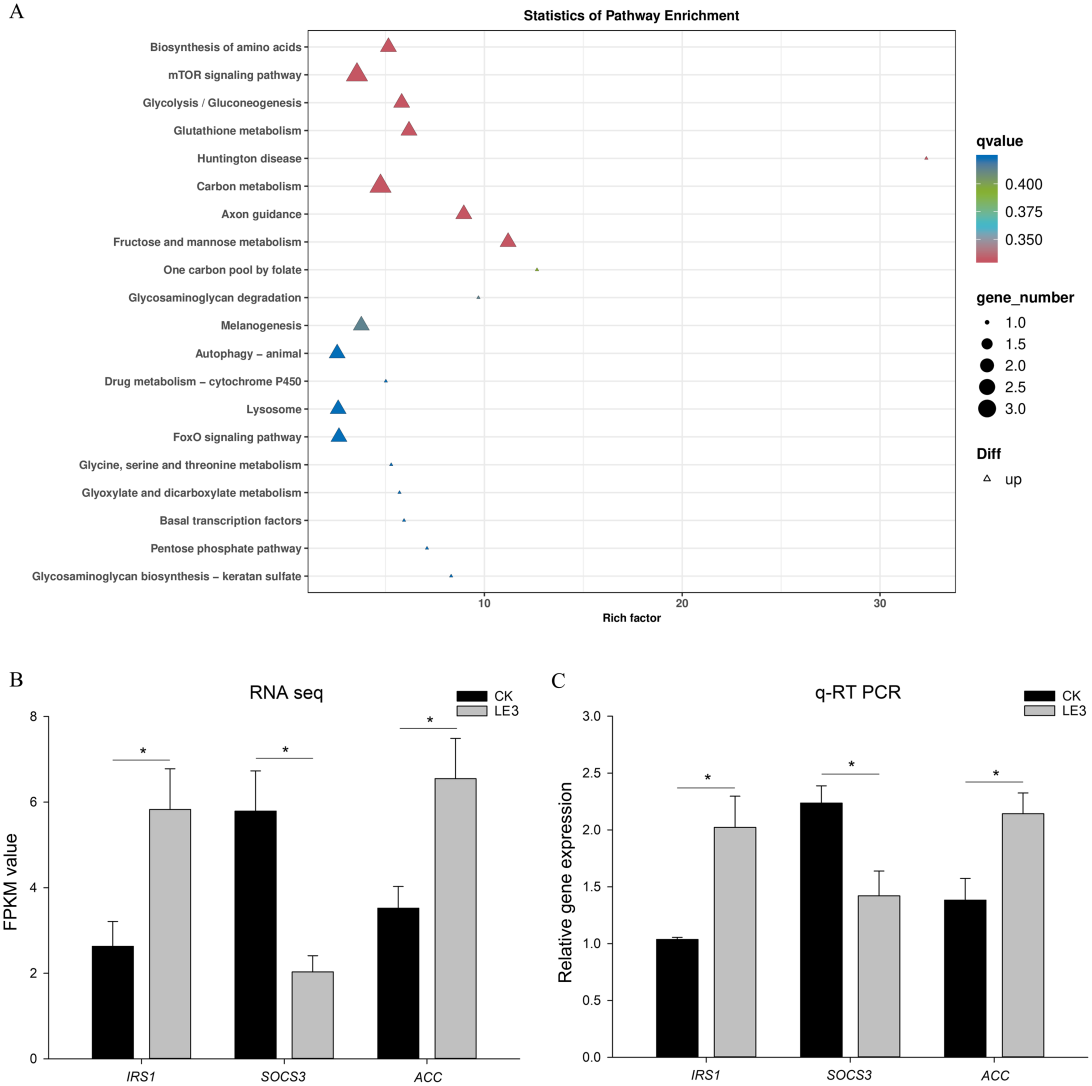
Comparative transcriptome analysis of liver tissue of tilapia fed a high leucine diet (2.4% leucine addition, LE3) versus a control diet (CK). **(A)** Enriched KEGG pathways of significantly differentially expressed genes (DEGs). **(B)** DEGs identified from RNA-Seq analysis. **(C)** Validation of the expression levels of DEGs in RNA-Seq data through qPCR analysis. qRT-PCR data were calculated by the 2^−ΔΔCt^ method with *β-Actin* as an internal control. Significance is denoted by an asterisk, with *p* < 0.05.

KEGG analysis of the differentially expressed genes (DEGs) (LE3 vs. CK) revealed enrichment of 20 pathways, including amino-acid biosynthesis, mTOR signaling, glycolysis/gluconeogenesis, Huntington disease, carbon metabolism, axon guidance, and fructose/ mannose metabolism (Figure 4A).

As mTOR signaling is associated with lipid metabolism, we validated three relevant genes by qRT-PCR (Figures 4B, C). It was observed that the expression levels of certain DEGs, such as *ACC* (acetyl-CoA carboxylase) and *IRS1* (insulin receptor substrate 1), showed a significant increase in response to high leucine levels (*p* < 0.05). Conversely, the expression level of *SOCS3* (suppressor of cytokine signaling 3) was found to be decreased in the group treated with leucine supplementation compared with the control group.

### 3.4 Leucine affects gut microbial community in tilapia

**Figure 5:**
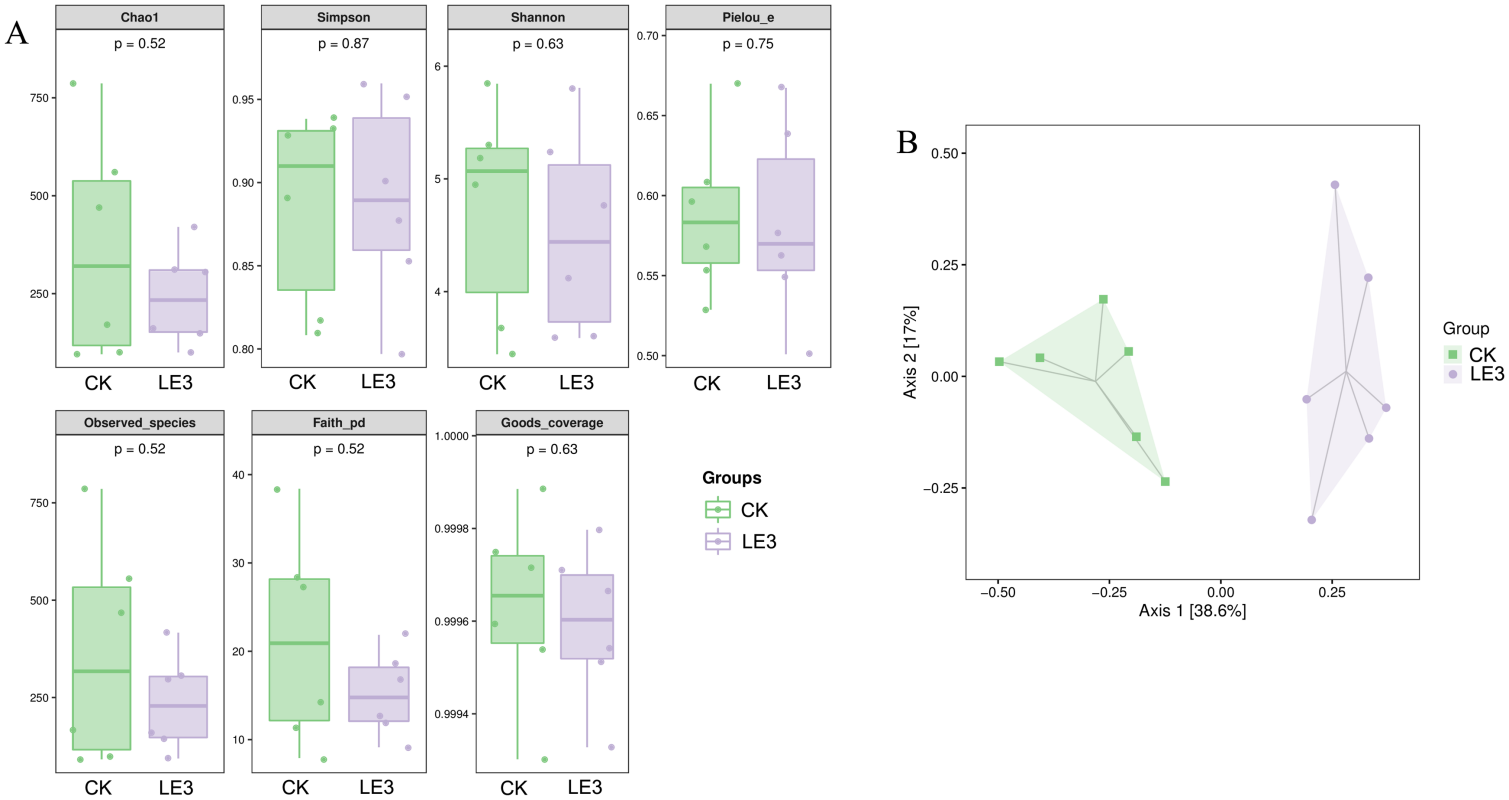
Alpha and beta diversity of gut microbiota of tilapia fed a high leucine diet (2.4% leucine addition, LE3) versus a control diet (CK). **(A)** Alpha diversity at the ASV level. **(B)** Principal coordinates analysis (PCoA) plot based on Bray-Curtis distances.

The analysis of α diversity, as determined by the Chao1 (*p* = 0.52), Simpson (*p* = 0.87), Shannon (*p* = 0.63), Plelou_e (*p* = 0.75), Observed_species (*p* = 0.52), Faith_pd (*p* = 0.52), and Good_coverage (*p* = 0.63) indices, did not reveal any significant difference in gut microbiota abundance between the LE3 and CK groups (*p* > 0.05) (Figure 5A). To further illustrate the variations in taxonomic composition between the two groups, we employed the PCoA analysis, which clearly demonstrated the distinct separation of samples from the LE3 and CK groups (Figure 5B).

**Figure 6:**
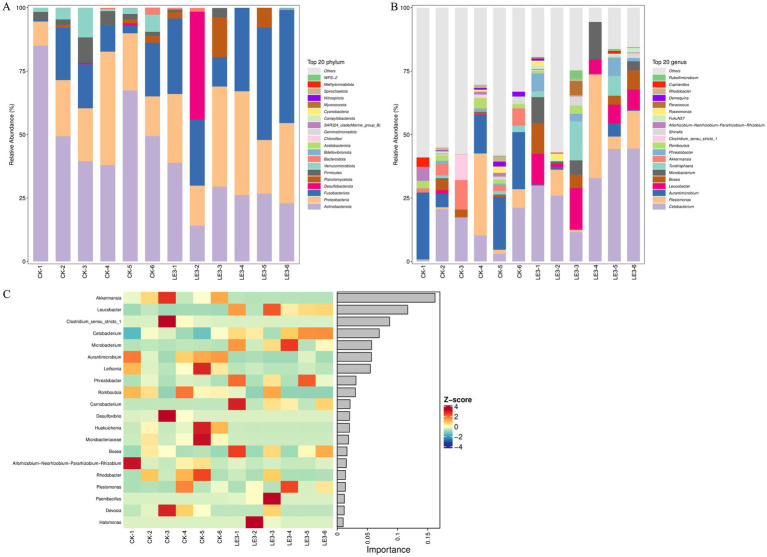
Gut microbiota composition of tilapia fed a high leucine diet (2.4% leucine addition, LE3) versus a control diet (CK) analyzed by 16S rRNA sequencing. **(A)** Taxonomic composition of the microbiota community at the phylum level. **(B)** Taxonomic composition at the genus level. **(C)** Top 20 genera ranked by importance in the random forest classification model.

In the CK group, Actinobacteriota (54.8%), Proteobacteria (22.6%), Fusobacteriota (12.2%), Verrucomicrobiota (4.6%), and Firmicutes (4.1%) were identified as the most abundant phyla in the intestine of tilapia. In LE3 group, the dominant phyla were Fusobacteriota (31.5%), Proteobacteria (29.3%), Actinobacteriota (26.3%), Desulfobacterota (7.1%), and Planctomycetota (4.2%) (Figure 6A).

At the genus level, intestinal microbiota was dominated by unclassified_Microbacteriaceae (37.8%), *Aurantimicrobium* (15%), *Cetobacterium* (12.1%), *Plesiomonas* (7.0%), and *Akkermansia* (4.5%) in the CK group, whereas it was dominated by *Cetobacterium* (31.5%), *Plesiomonas* (12%), unclassified_Microbacteriaceae (10.4%), *Leucobacter* (8.5%), and unclassified_Desulfovibrionaceae (7.1%) in the LE3 group (Figure 6B).

Furthermore, to elucidate the taxa contributing to the observed differences between the intestinal microbiomes of the LE3 and CK groups, random-forest classification was employed using genus-level relative abundance data. The top 20 discriminatory genera were identified. The results revealed that genera *Akkermansia*, *Leucobacter*, *Clostridium*_sensu_stricto_1, *Cetobacterium*, and *Microbacterium* exerted the highest influence (Figure 6C). Notably, *Leucobacter* and *Cetobacterium* exhibited an increase in LE3 group relative to CK group (Figures 6B,C).

### 3.5 NK01 supplementation improves growth and serum biochemical parameters in tilapia

**Figure 7:**
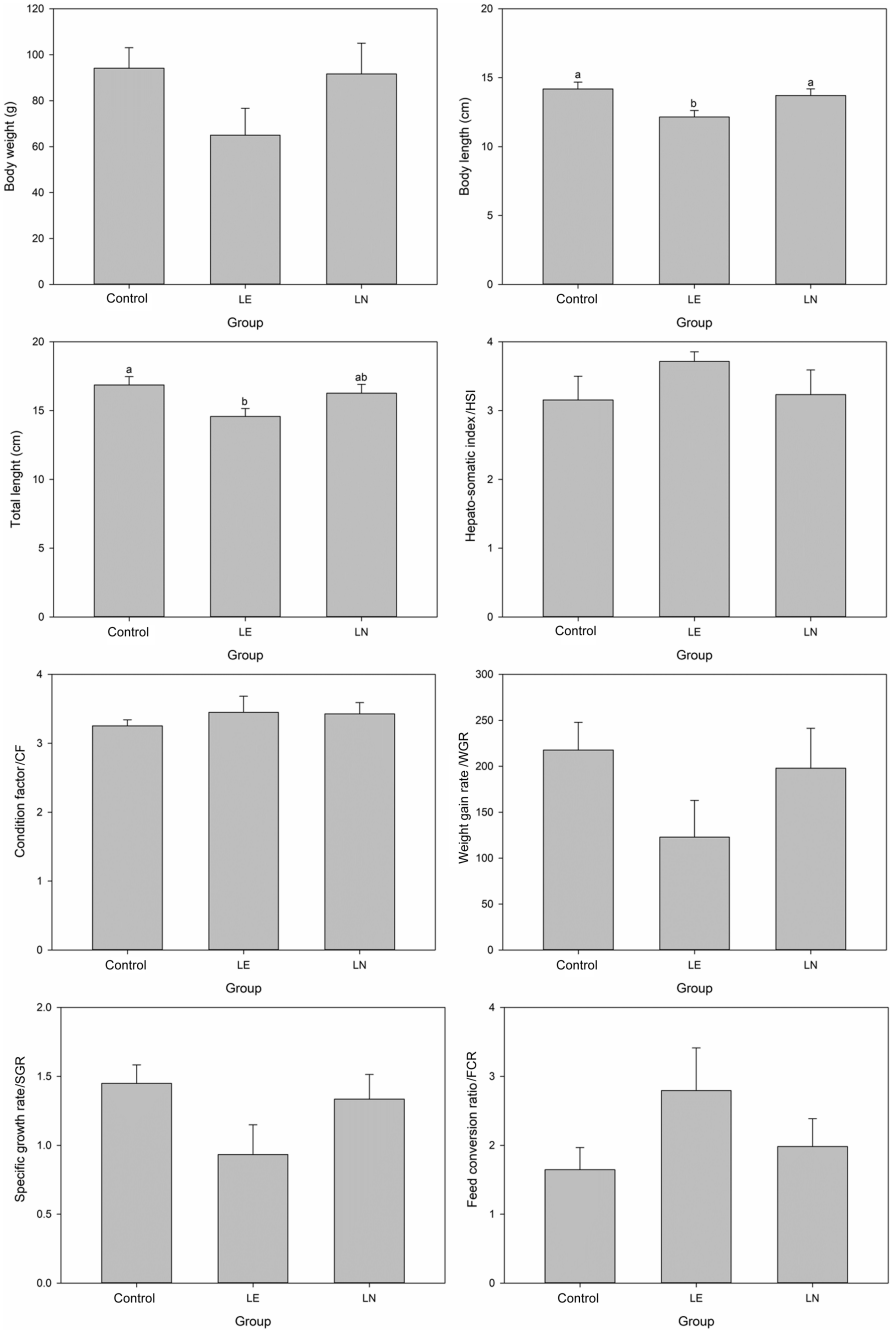
Growth performance parameters of tilapia fed diets containing different levels of leucine, with or without *Cetobacterium* NK01 supplementation. LE: 2.4% leucine addition, LN: 2.4% leucine addition + *Cetobacterium* NK01-supplement. Different lowercase letters indicate significant differences among groups for the same parameter (*p* < 0.05).

The body and total length of tilapia were significantly shorter in the 2.4% leucine-induced fatty liver (LE) group than those in the control diet (CK) group and the 2.4% leucine-induced fatty liver treated with the *C. somerae* NK01 supplement (LN) group (*p* < 0.05) (Figure 7). Body weight, WGR and SGR were also lower in the LE group, but the differences were not significant (*p* > 0.05). HSI, CF and FCR were numerically higher in the LE group, without significance (*p* > 0.05).

**Figure 8:**
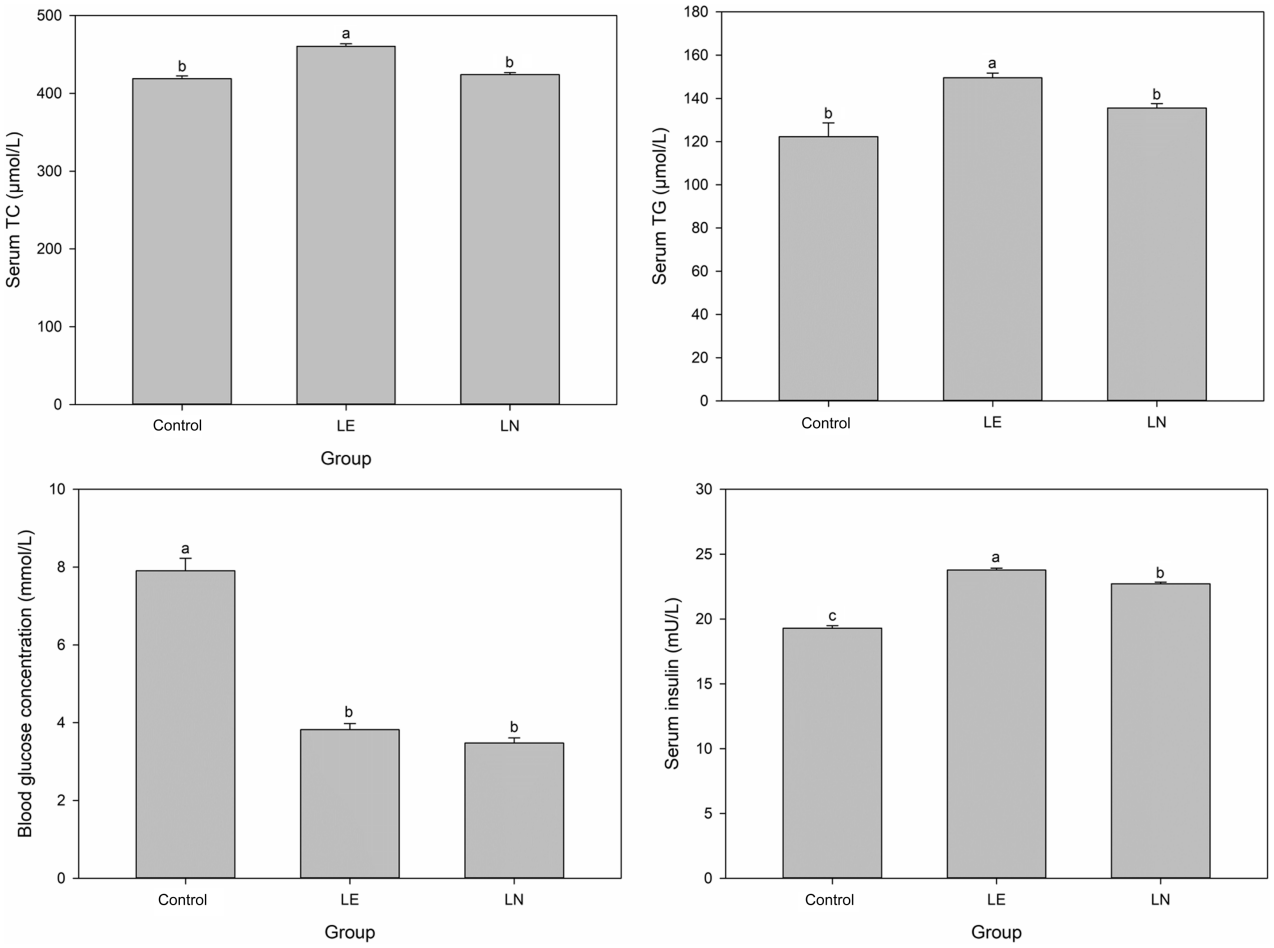
Levels of TC, TG, glucose, and insulin in the serum of tilapia fed diets containing different levels of leucine, with or without *Cetobacterium* NK01 supplementation. LE: 2.4% leucine addition, LN: 2.4% leucine addition + *Cetobacterium* NK01-supplement. Different lowercase letters indicate significant differences among groups for the same parameter (*p* < 0.05).

Significant differences were observed in the serum TC, TG and insulin of tilapia in the LE group compared with the other two groups (*p* < 0.05). Serum TC, TG and insulin were markedly elevated in the LE group compared with the CK and LN groups (*p* < 0.05) (Figure 8). Blood glucose was highest in the CK group and significantly higher than in the LE and LN groups (*p* < 0.05).

### 3.6 Hepatoprotective effect of NK01 against leucine-induced steatosis

**Figure 9:**
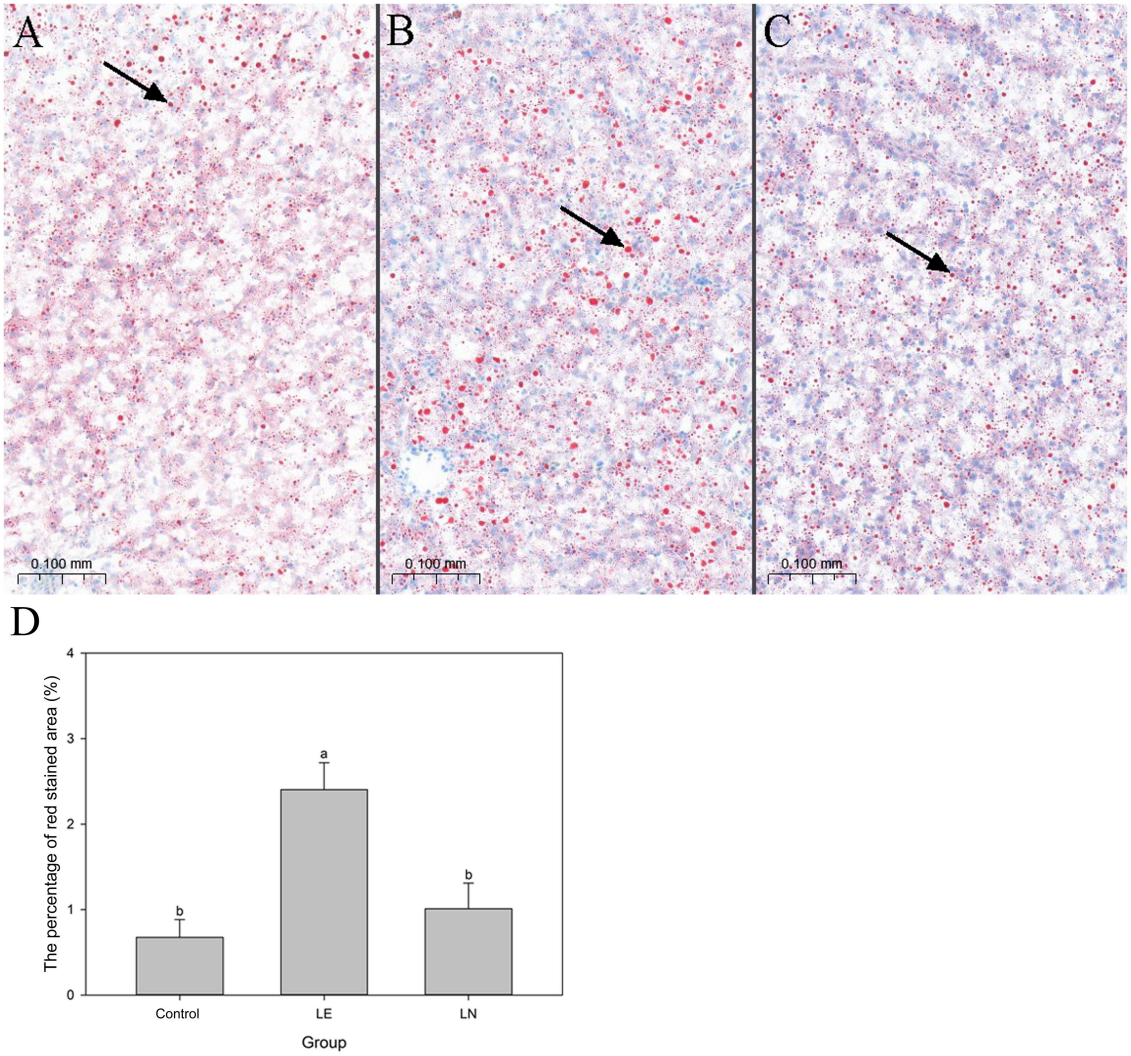
Oil-Red O staining of hepatic lipid droplets in tilapia fed diets containing different levels of leucine, with or without *Cetobacterium* NK01 supplementation. **(A)** Oil-Red O staining of hepatic lipid droplets in the control group. **(B)** Oil-Red O staining of hepatic lipid droplets in the LE group. **(C)** Oil-Red O staining of hepatic lipid droplets in the LN group. **(D)** The percentage of red stained area quantified utilizing ImageJ Pro Plus 6 software. Original magnification 40×; scale bars: 0.05 mm. Lipid droplets are displayed in red; nuclei in blue. LE: 2.4% leucine addition, LN: 2.4% leucine addition + *Cetobacterium* NK01-supplement.

The tissue stained with Oil Red O revealed a higher degree of steatosis in the LE group in comparison to the CK group, while a reduction in steatosis was observed in the LN group as compared with the LE group (Figures 9A–C). Furthermore, the relative lipid-droplet area was significantly increased in the LE group when compared with the CK group (*p* < 0.05) (Figure 9D). The LN group exhibited a significantly lower relative lipid-droplet area when compared with the LE group (*p <* 0.05) (Figure 9D).

### 3.7 NK01 administration regulates serum amino-acid profiles and SCFAs levels of gut content of tilapia

**Figure 10:**
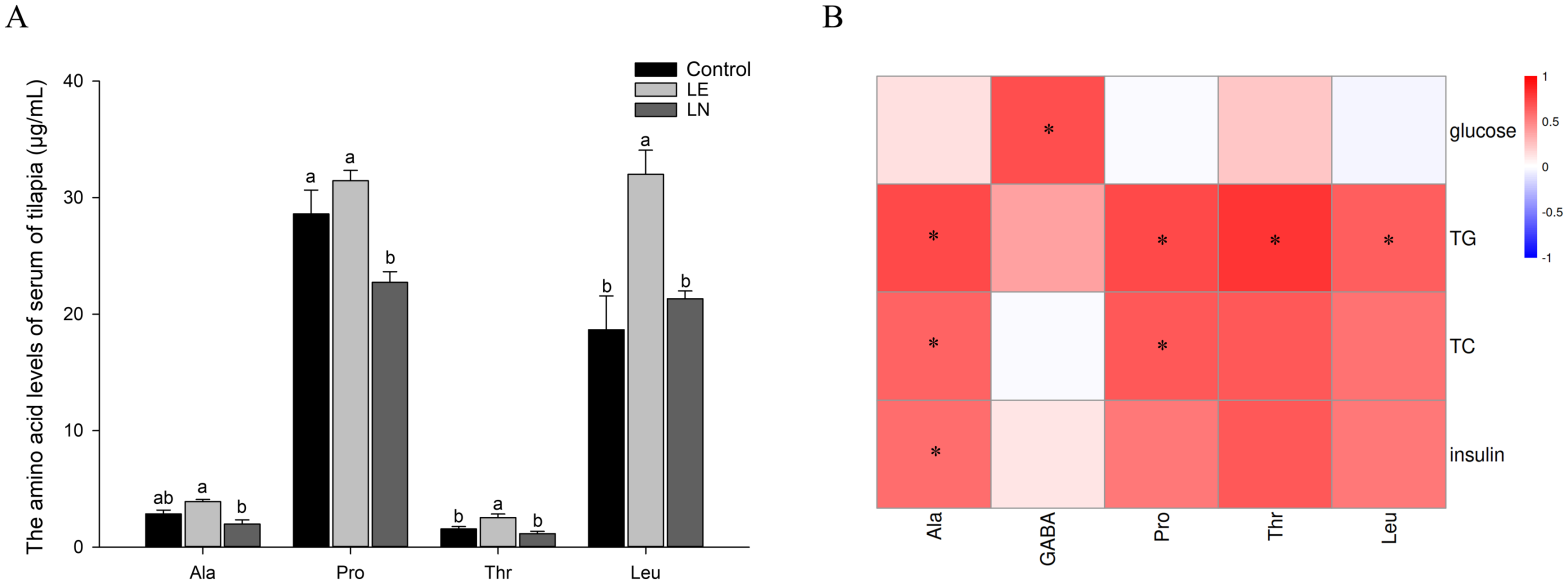
Targeted metabolomic analysis of serum amino acids of tilapia. **(A)** The amino acid levels. Different letters indicate significant differences among groups for the same amino acid (*p* < 0.05). **(B)** Heatmap of Pearson correlations between biochemical parameters and the five differential amino acids comparing LN with LE. Red = positive correlations; blue = negative correlations. Significance is denoted by an asterisk, with *p < 0.05 in the heatmap. LE: 2.4% leucine addition, LN: 2.4% leucine addition + *Cetobacterium* NK01-supplement. Ala: alanine; Pro: proline; Thr: threonine; Leu: leucine; GABA: gamma-aminobutyric acid.

Compared with the CK group, the levels of serum leucine and threonine were significantly higher in the LE group (*p* < 0.05), whereas alanine and proline showed no significant change (*p* > 0.05) (Figure 10A). The LN group showed significantly lower levels of alanine, proline, threonine, and leucine relative to the LE group (*p* < 0.05).

To investigate the relationship between amino acids and biochemical parameters, we conducted a Pearson rank correlation test. The test result revealed that a total of five differential amino acids exhibited significant correlations with at least one biochemical parameter (Figure 10B). Alanine, proline, threonine and leucine were positively correlated with TG (*p* < 0.05); alanine and proline were also positively correlated with TC (*p* < 0.05). Gamma-aminobutyric acid (GABA) showed a positive correlation with glucose (*p* < 0.05).

**Figure 11:**
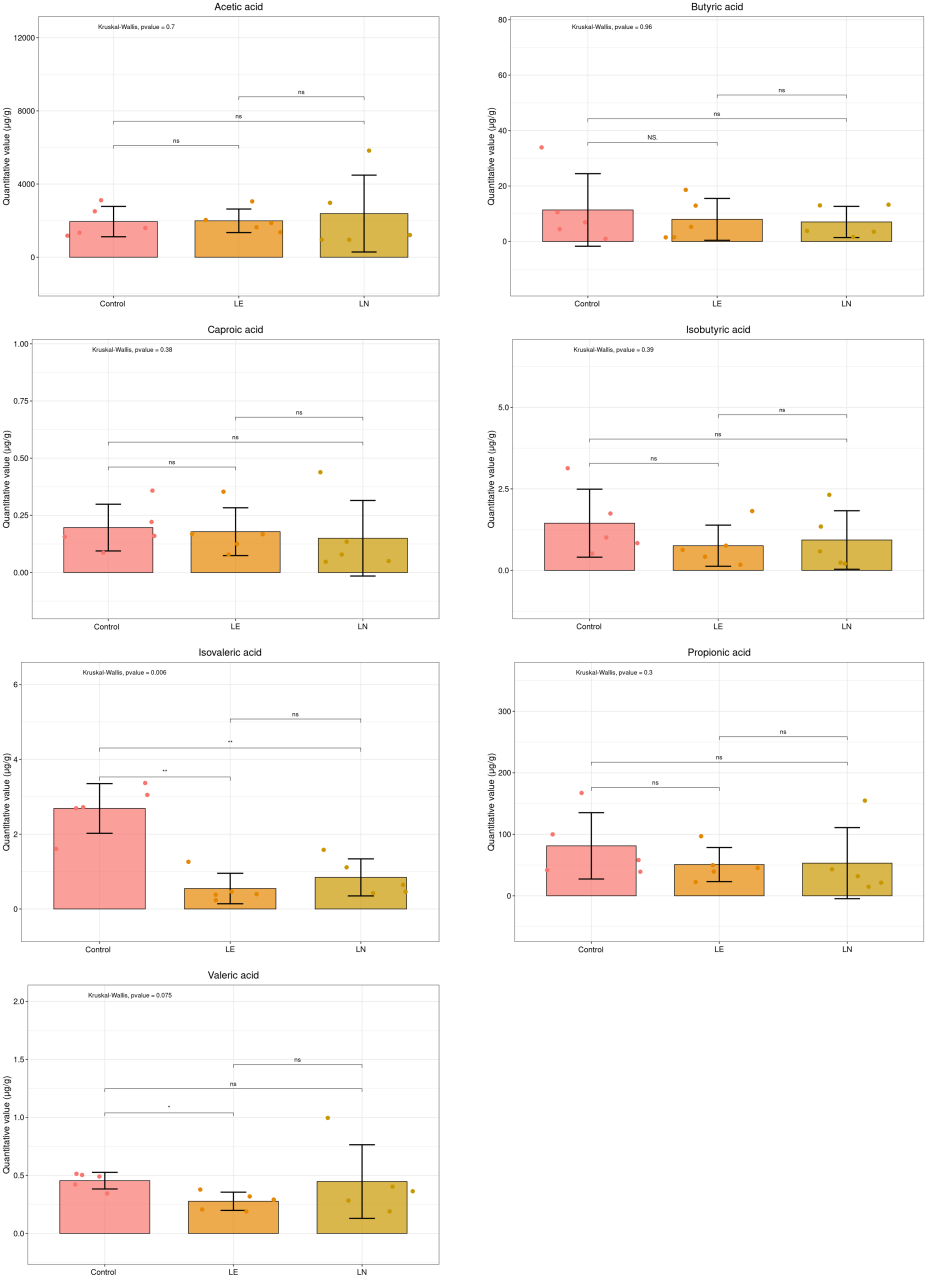
The short chain fatty acid (SCFA) levels of gut content. LE: 2.4% leucine addition, LN:2.4% leucine addition + *Cetobacterium* NK01-supplement. *p < 0.05, **p < 0.01. “ns” indicates no significant difference (*p* > 0.05).

The concentrations of SCFAs of the gut content are shown in Figure 11. Valeric acid exhibited a notable reduction in the LE group in comparison to the CK group (*p* < 0.05). Isovaleric acid displayed a significant decrease in both the LE and LN groups when compared with the CK group (*p* < 0.05). However, no statistically significant differences were detected for acetic acid, butyric acid, caproic acid, isobutyric acid, and propionic acid among the three groups (*p* > 0.05).

### 3.8 NK01 modulates lipid-metabolism gene expression

**Figure 12:**
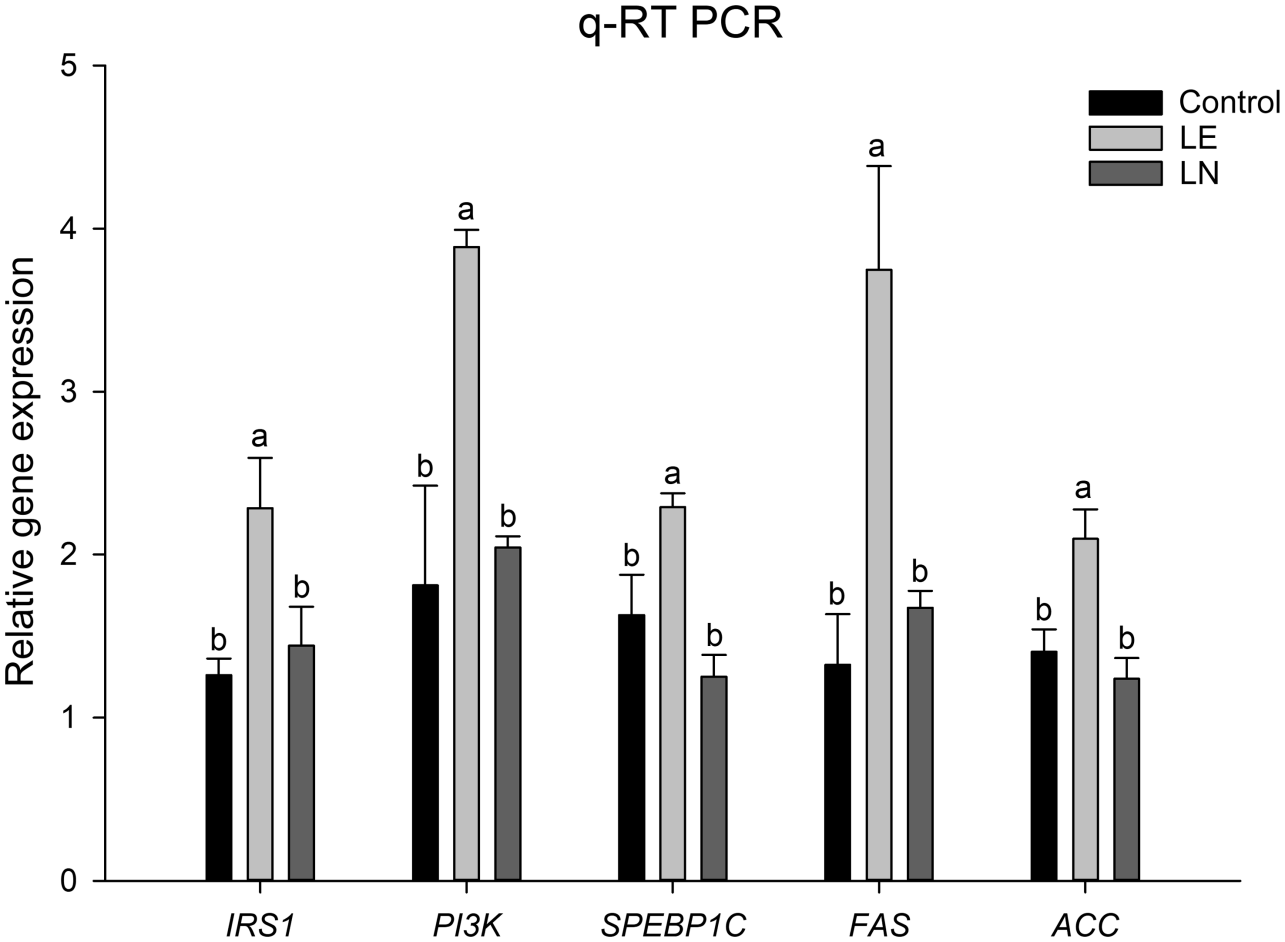
Relative mRNA expressions of genes involved in lipid-metabolism signaling pathway. LE: 2.4% leucine addition; LN: 2.4% leucine addition + *Cetobacterium* NK01-supplement. Different lowercase letters indicate significant differences among groups for the same gene (*p* < 0.05).

The mRNA expression levels of *IRS1*, *PI3K* (phosphoinositide 3-kinase), *SPEBP1C* (sterol regulatory element-binding protein-1C), *FAS* (fatty acid synthase), and *ACC* genes were assessed. Transcript levels of *IRS1*, *PI3K*, *SREBP1c*, *FAS* and *ACC* were significantly elevated in the LE group compared with the CK group (*p* < 0.05). The LN group displayed a significant reduction in transcript levels for *IRS1*, *PI3K*, *SPEBP1c*, *FAS*, and *ACC* genes when compared with the LE group (*p* < 0.05) (Figure 12).

## 4 Discussion

Nile tilapia is an omnivorous fish species known for its high tolerance to dietary carbohydrates, with level ranging from 38 to 46% (corresponding to 7.0–7.38% dietary lipid) (Wang et al., 2005). In order to achieve the lowest cost production to meet global demand, nutritional studies have increasingly focused on developing cost-effective feeds with high carbohydrate content for tilapia aquaculture (Boonanuntanasarn et al., 2018). However, the shift toward plant-based feed ingredients has introduced various anti-nutritional factors and digestive challenges (Gule and Geremew, 2022). The present study observed hepatic fat accumulation in the control group, likely attributable to the high carbohydrate content commonly found in commercial tilapia feeds. It has been reported that elevated blood glucose level can lead to lipid deposition in fish. For instance, Nile tilapia fed a diet containing 45% corn starch exhibited higher plasma TG level and greater hepatic vacuolation area compared to those fed 30% corn starch. This high-starch diet also induced signs of liver damage, as evidenced by significantly elevated plasma aspartate aminotransferase (AST) and alanine aminotransferase (ALT) activities (Li L. Y. et al., 2021). Similarly, another study reported that a 45% corn starch diet significantly up-regulated the expression of lipogenic genes such as *DGAT* (diacylglycerol acyltransferase), *SREBP1*, and *FAS*, and induced fatty liver formation in Nile tilapia (Zhu et al., 2020).

In murine models, leucine supplementation has been shown to alleviate hepatic steatosis under high-fat dietary condition (Jiao et al., 2016); however, these metabolic benefits were less evident in obese animals (Binder et al., 2013). When leucine was co-ingested with glucose, it attenuated the postprandial glucose response and enhanced insulin secretion in human (Kalogeropoulou et al., 2008). In the present study, leucine-supplemented groups enhanced insulin secretion and reduced blood glucose levels in tilapia. This effect may be mediated by leucine-induced activation of the mTORC1 (mechanistic target of rapamycin complex 1) pathway in pancreatic β cells, as well as through its metabolites. Human studies have similarly demonstrated that leucine promoted insulin secretion via multiple signaling pathways (Yang et al., 2010). In largemouth bass (*Micropterus salmoides*), dietary leucine supplementation significantly increased the expression of *IRS1* (Shao et al., 2024). However, despite its hypoglycemic effect, leucine supplementation did not mitigate carbohydrate-induced fatty liver in this study. Instead, it led to elevated serum TC and TG levels. Leucine is a ketogenic amino acid, whose carbon skeleton is converted into acetyl-CoA and acetoacetic acid in tissues, and these intermediates can be used for fatty acid synthesis (Zehra and Khan, 2015). This is consistent with previous findings indicating that dietary leucine level was positively correlated with lipid metabolism in fish. Elevated leucine intake has been shown to upregulate the expression of key lipogenic genes such as *FAS*, *ACC*, and to increase serum TG levels and body fat content. For example, in Indian major carp fingerling, carcass fat showed a positive trend with increasing dietary leucine concentration (Ahmed and Khan, 2006; Zehra and Khan, 2015). Similarly, juvenile blunt snout bream fed a diet containing 1.33% leucine exhibited higher serum TG level and hepatic expression of lipogenic enzyme genes compared to those fed 0.90% leucine (Liang et al., 2019). Collectively, these results suggested that while leucine enhanced insulin secretion and lowered blood glucose, it exacerbated hepatic lipid accumulation and may inhibit growth under high-carbohydrate dietary condition in fish.

In this study, leucine supplementation enhanced amino acid biosynthesis and activated the mTOR signaling pathway in the liver of tilapia. Porstmann et al. (2008) demonstrated that *mTORC1* plays a crucial role in the positive regulation of *SREBP1*, a transcription factor responsible for controlling the expression of genes involved in lipid metabolism, such as *FAS*. In rainbow trout hepatocytes, upregulation of *FAS* gene expression was closely associated with activation of the TOR pathway and the increased *SREBP1* level (Lansard et al., 2010). In the present study, the addition of leucine to the feed of tilapia resulted in a significant upregulation of *SREBP1c*, *ACC*, and *FAS* genes in liver, indicating that leucine activated the TOR pathway and promoted lipid synthesis.

Leucine-induced alterations in lipid metabolism may also be mediated by the gut microbiota (Hu et al., 2019), as intestinal microbiota can influence leucine absorption and metabolism in enterocytes (Hu et al., 2017). By analyzing the impact of leucine on the intestinal microbiota structure of tilapia, we found that the relative abundance of two dominant bacteria significantly increased. Among them, *Cetobacterium* was the core bacterium of tilapia (Zhang et al., 2022). Previous studies have reported that *Cetobacterium* or its fermentation products can significantly reduce serum glucose, TC, and TG levels in tilapia (Wang et al., 2021; Xie et al., 2022; Xie et al., 2021). Moreover, our previous work demonstrated that *C. somerae* strain NK01 could attenuate lipid synthesis in tilapia (Wang et al., 2025). Therefore, we hypothesized that *C. somerae* NK01 might alleviate leucine-induced metabolic disorders. We found that co-supplementation with leucine and *Cetobacterium* decreased the leucine levels in the serum of tilapia. Correlation analysis revealed a significant positive relationship between serum leucine and TG levels, suggesting that *Cetobacterium* may alleviate leucine-induced hepatic lipid accumulation by metabolizing leucine. Studies have found that *Cetobacterium* lowered tilapia blood glucose and stimulated insulin secretion by raising intestinal acetic acid level in zebrafish (Wang et al., 2021). Here, we quantified SCFAs in tilapia intestinal content and found that combined addition of *Cetobacterium* and leucine did not significantly increase acetic acid concentration. This suggested that the glucose-lowering effect of *Cetobacterium* may not be mediated by acetate production.

## 5 Conclusion

This study revealed the dual role for leucine in lipid metabolism: it promoted lipogenesis through the mTOR-SREBP1c axis, while the intestinal microbe *Cetobacterium* may partially alleviate this effect. The findings pointed to a potential function of *Cetobacterium* in modulating leucine metabolism and subsequent hepatic lipid deposition.

## Data Availability

The datasets presented in this study can be found in online repositories. The names of the repository/repositories and accession number(s) can be found at: https://www.ncbi.nlm.nih.gov/, PRJNA1330518; https://www.ncbi.nlm.nih.gov/, PRJNA1354571.
