# Supplementary material for: Cetobacterium alleviates the lipid accumulation in Nile tilapia (Oreochromis niloticus) induced by leucine addition
Source: Front Microbiol. 2026 Jan 16;16:1708010. doi: 10.3389/fmicb.2025.1708010 (PMC12858182; doi:10.3389/fmicb.2025.1708010)
Supplement: Supplementary file 1 [file Supplementary_file_1.docx]

Supplemental Table 1 Ingredients of the diet (dry matter, g/kg diet)

| Ingredient (g/kg diet) | Dry matter |
| --- | --- |
| Fish meal | 50 |
| Soybean meal | 300 |
| Rapeseed meal | 225 |
| Wheat starch | 370 |
| Soybean oil | 20 |
| Vitamin premix^1^ | 10 |
| Mineral premix^2^ | 10 |
| Monocalcium phosphate | 12 |
| Choline chloride | 3 |
| Total | 1000 |
| Ash | 8% |
| Crude protein | 23% |
| Crude lipid | 6% |
| Crude fiber | 10% |

^1^ Containing the following (/kg vitamin premix): Vitamin A, 10,000 I.U.; Vitamin D, 1500 I.U.; Vitamin E, 0.1 g; Vitamin C, 0.5 g; thiamine, 2.0 g; riboflavin, 0.9 g; pyridoxine HCl, 0.1 g; vitamin B-12, 0.9 g; menadione sodium bisulfite, 0.1 g; retinyl acetate, 0.6 g; cholecalciferol, 0.1 g; dl-α-tocopherol-acetate, 0.1 g.

^2^ Containing the following (g/kg mineral premix): CuSO_4_∙5H_2_O, 1.2; FeSO_4_∙7H_2_O, 6.5; MnSO_4_∙H_2_O, 1.5; KIO_3_, 0.3; ZnSO_4_∙7H_2_O, 1.2; Ca(H_2_PO_4_)_2_∙2H_2_O, 0.1.

Supplemental Table 2 Primer sequences

| Primer name | Forward sequence (5' to 3') | Reverse sequence (5' to 3') | Accession numbers |
| --- | --- | --- | --- |
| *FAS* | TCATCCAGCAGTTCACTGGCATT | TGATTAGGTCCACGGCCACA | XM_003454056.5 |
| *ACC* | GGAGTTCGGACAGCACCTATGAA | AGCAGGAGAAGCAACAGTGAAGT | XM_025910659.1 |
| *SREBP1c* | ATTCAGACTCAGCTCCAAGGG | TCAGGCTTCAAAGTGGTCAG | XM_005457771.4 |
| *β-Actin* | CAGCAAGCAGGAGTACGATGAG | TGTGTGGTGTGTGGTTGTTTTG | XM_003443127.5 |
| *PI3K* | CCAAAACCACTACCTCTGCG | TCGTTCTTCATCAATGCCAA | XM_019358209.2 |
| *IRS1* | GCCTCCTTACCTCCTATG | GGCTCGTGCTTCTTGACA | XM_025905568.1 |
| *SOCS3* | ACCCTCAGTGTCAAGACAGCCTC | AGAACGCAGTCAAAGTGGGGAA | NM_001319870.1 |

*FAS* (fatty acid synthase), *ACC* (acetyl-CoA carboxylase), *SPEBP1C* (sterol regulatory element-binding protein-1C), *PI3K* (phosphoinositide 3-kinase), *IRS1* (insulin receptor substrate 1), *SOCS3* (suppressor of cytokine signaling 3)
